# Adenoviral Conjunctivitis in the Andaman Islands: A Clinical and Molecular Epidemiological Study

**DOI:** 10.7759/cureus.51241

**Published:** 2023-12-28

**Authors:** Nisha Beniwal, Rehnuma Parvez, Baljeet Saharan, Vineeta Malik, Rahul Dhodapkar, Nagarajan Muruganandam

**Affiliations:** 1 Infectious Disease, Indian Council for Medical Research - Regional Medical Research Centre, Port Blair, IND; 2 Microbiology, Chaudhary Charan Singh Haryana Agricultural University, Hisar, IND; 3 Ophthalmology, Dr Agarwals Eye Hospital, Port Blair, IND; 4 Infectious Disease, Jawaharlal Institute of Postgraduate Medical Education and Research, Puducherry, IND

**Keywords:** molecular sequencing, epidemiology, andaman, adenovirus, : keratoconjunctivitis

## Abstract

Introduction

Human adenoviruses are common causes of many acute illnesses, and keratoconjunctivitis is one of them. Acute infections, if left untreated, can progress to severity, thus causing morbidities and mortalities. It belongs to the mastadenovirus family and is characterized by seven subgenus, i.e., A-G; among those, Adenovirus D8 is the most common type associated with keratoconjunctivitis.

Methodology

A hospital-based study was conducted, and the samples were collected from GB Pant Hospital, Port Blair, Dr Agarwals Eye Hospital, Port Blair, from August 2017 to December 2022. Clinical data and demographic details were followed by conjunctival swab sample collection from suspected keratoconjunctivitis patients. Samples were subjected to molecular screening, and Sanger sequencing was carried out for positive samples.

Results

Out of 506 conjunctival samples, a prevalence of 24.9% (n=126) was observed, and the commonest type circulating among the population of Andaman was Adenovirus D8. The major symptoms associated were eye redness (87.30%, n=110), followed by watering (81.75%, n=103), eye pain (72.22%, n=91), eye itching (61.11%, n=77), and discharge (50%, n=63).

Conclusion

In clinical research, ocular infections are one of the underrated fields. However, the study revealed the high prevalence of adenoviral infection among the suspected patients. Thus, there is a need for proper surveillance and timely diagnosis of such infections, as their severity may lead to loss of vision.

## Introduction

Adenoviruses are a common cause of many illnesses, including the typical flu and cold: fever, throat discomfort, red eyes (conjunctivitis), acute bronchitis, pneumonia, and acute gastrointestinal (GI) disease, causing diarrhea, vomiting, and nausea [1]. Adenoviruses can also cause neurologic diseases, such as those that affect the brain, spinal cord, and bladder, but are infrequent.

The human Adenovirus (HAdV) is a member of the Adenoviridae family comprising non-enveloped viruses. The diameter of the discovered entities spans 65 to 80 nm. The virion consists of a protein capsid composed of 252 capsomeres. The nucleoprotein core contains the viral DNA. The viral DNA is double-stranded and ranges in length from 26 to 46 kilobase pairs (kbp)[2]. The viral capsid has icosahedral symmetry and comprises three major proteins: hexon, penton base, and fiber. Four minor proteins, namely IIIa, VI, VIII, and IX, are associated with major proteins. The capsid protein known as hexon exhibits the largest size and highest abundance within the viral shell. One capsid facet is composed of 20 hexon homotrimers. The foundation of the spike on the vertex is comprised of trimers of glycosylated fiber proteins. The homopentamers of penton-base proteins are present on each edge of the icosahedral structure [2,3]. The International Committee on Taxonomy of Viruses (ICTV) has categorized human adenovirus (HAdV) into seven subgenera: A-G. Phylogenetically, a comprehensive set of 90 genotypes has been recognized based on all the genome sequences available in the GenBank database. Among these genotypes, 51 serotypes have been identified by a serum neutralization assay [3].

Typically, acute respiratory illness is caused by adenovirus types 3, 4, and 7. Enteric adenovirus types 40 and 41 are known to produce diarrhea, a typical symptom of gastroenteritis in young children. Adenovirus 8, 19, 37, 53, and 54 are responsible for the widespread kerato-conjunctivitis outbreaks [4,5]. Up to 90% of viral conjunctivitis is caused by adenoviruses, making them the most common cause of infectious conjunctivitis globally. The most members and the strongest relationship with viral conjunctivitis are found in the HAdV-type D species [6].

Shaking hands is one of the main transmission modes through direct skin-to-skin contact. While touching an infected object or surface and then touching one's mouth, nose, or eyes is an example of indirect skin-to-air contact. Diaper changes are also common settings for transferring Adenovirus by feces from one person to another. Adenovirus can occasionally be spread by water; man-made swimming pools are responsible for the potential risk associated with Adenovirus due to their ability to remain dormant on inanimate surfaces and objects for extended periods. HADV stability is notoriously challenging to eradicate. The Adenovirus may still be transmitted through "viral shedding" even without apparent symptoms [1,7].

The Andaman and Nicobar Islands are located remote from the Indian mainland and are a union territory of India with a population of 4,34,192 as of 2019 [8]. Therefore, this investigation aims to perform a molecular characterization of HAdV identification and types circulating in this population by analyzing the molecular assays. Viral keratoconjunctivitis that is, Adenoviral keratoconjunctivitis, Herpes Simplex keratoconjunctivitis, and Enteroviral keratoconjunctivitis are the major contributors and can lead to permanent vision loss if left untreated. Furthermore, the study and findings connected to Adenovirus were driven by the dearth of data available on the human adenoviruses (HAdVs) associated with keratoconjunctivitis.

## Materials and methods

Ethical Approval

Prior to starting the investigation, ethical clearance was acquired from the institutional ethical board (RMRC-29/06/2017/06).

Study type, study duration, and sample size

A hospital-based study was conducted, and the samples were collected from GB Pant Hospital, Port Blair, and Dr Agarwals Eye Hospital, Port Blair. The inhabitants of the Andaman Islands were the study population in this study. A pre-structured questionnaire was completed, and samples were collected after obtaining informed written consent from each study participant. A total of 506 conjunctival swab specimens were collected in viral transport media(VTM) for screening for adenoviral presence in patients from August 2017 to December 2022. Clinical manifestations of redness of the eye with serous and mucoid discharge were considered for collection of ocular swab specimen collection.

DNA extraction from conjunctival swab samples

Screening was carried out primarily by following three steps to detect and confirm HAdV in suspected conjunctival samples. During the study period, conjunctival swab specimens were collected in VTM from the patients presenting to the eye care setting suspected of keratoconjunctivitis in the Andaman Islands. A total genomic DNA was subjected by using the Pure Link Viral RNA/DNA Mini Kit (Invitrogen, USA, cat no. 12280-050) as per the manufacturer's instructions (including negative controls). A 45µl of DNA was finally eluted and stored at -20°C for further use.

PCR reaction mix and thermal cycling conditions for detecting adenovirus in conjunctival specimens

The standard PCR method was used to amplify genomic DNA from conjunctival samples to detect the presence of adenoviral DNA. The final reaction volume of 25 µl contained 12.5 µl of 2X PCR master mix (Cat no. K0171), 7 µl of template DNA, 0.7 µl of each primer (forward primer -5'-CTGTGGTCGACTTGCAAGAC-3'; and reverse primer -5'-ACCGCAGAGTTCCACATACT-3'), and 4.1 µl of nuclease-free water, yielding a 102 bp amplification product [9]. The following cycling conditions were used to perform the amplification on an Applied Biosystems Veriti 96-well thermal cycler (Model No. 9902, Singapore): initial denaturation for 5 minutes at 95°C, followed by 30 seconds at 95°C, 30 seconds at 60°C for extension, for a total of 40 cycles and a final extension of 7 minutes at 72°C. Every PCR experiment had a negative and a positive control to ensure the results were reliable. After amplification, 10 µl of PCR products were separated into 2% agarose gel electrophoresis at 2%. The amplified adenovirus fragment was compared to a 50- and 100-bp DNA ladder. In addition, retrospective confirmation was carried out for the positive samples using a specific set of primers.

Another amplification with the hexon gene was carried out using a region-specific Adenovirus primer with an amplification range of 1092 bp. It was achieved by using a final reaction volume of 25µl containing 12.5µl of 2X PCR master mix (Cat no. K0171), 7μl of template DNA, 0.7µl of each primer (forward primer -5’-TGGAACTTCCGCAAGGA-3'; and reverse primer -5’-GGAGAAGGGCGTGCGCA-3'), and 4.1µl of nuclease-free water [10]. The amplification was carried out using the following cyclic conditions on an Applied Biosystems (Model No. 9902, Singapore) veriti 96-well thermal cycler: Denaturation for 80 seconds at 94^o^C, annealing for 40 seconds at 45^o^C, and Extension at 72^o^C for 90 seconds up to 40 cycles, followed by a final extension for 7 minutes. Each PCR experiment also included negative and positive controls to ensure accurate results. Following amplification, 10µl of PCR products were run on a 1% agarose gel electrophoresis to look for the adenovirus-amplified fragment against a 100-bp DNA ladder.

Out of the total positives from both experimental protocols, we were successfully able to type nine adenovirus-positive samples from each category by using Sanger's sequencing (Big Dye Chain Termination Method), and the raw sequences obtained were analyzed using Mega Software (Mega Version 11.0). The sequences were aligned and analyzed against worldwide reference sequences from the Gen-bank Database. Gen-bank reference sequences for Adenovirus were retrieved and used in the phylogenetic analysis to determine the circulating strain in the study area. The ClustalW program, included in the Mega software version 11.0, aligned Gen-bank sequences.

## Results

Association of Adenovirus among conjunctival specimens

**Figure 1:**
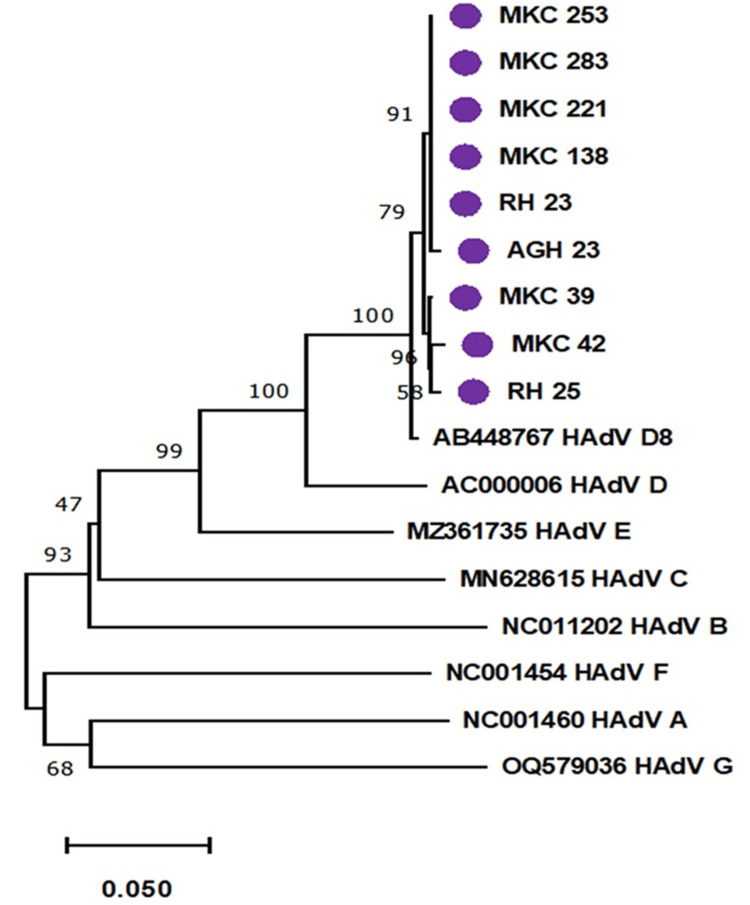
Neighbor-Joining bootstrap consensus phylogenetic tree analysis of Conjunctival Samples with reference sequences of Adenovirus (Hexon region)

In order to determine the prevalence of Adenovirus associated with Kerato-conjunctivitis, a total of 126 (24.9%) samples were found to be positive for Adenovirus. Almost all the patients who tested positive for Adenovirus showed symptoms within a week of testing (116, 92.06%). The most commonly affected age group was 31-40 years, i.e., 30 cases (23.89%), followed by 21-30 (26 cases, 20.63%) and 41-50 (22 cases, 17.46%) years. Compared to the diagnosed females, 70 males (55.56%) were observed more during the study period. Approximately 70% of cases had unilateral eye involvement, with more than half recorded to have left eye involvement (45.29% out of all positive cases). Frequently observed symptoms were eye redness (110; 87.30%), followed by watering (103; 81.75%), eye pain (91 cases; 72.22%), eye itching (77 patients; 61.11%), and discharge (63 cases; 50%). Only 5.5% of cases had a history of fever. Systematic infections like glaucoma, cataracts, hypertension, diabetes mellitus, and thyroid were not observed among the cases. The neighbor-joining bootstrap consensus phylogenetic tree (Figure 1) analysis revealed that the Adenovirus associated with conjunctivitis reported in the Andaman Islands had 100% genetic similarity with the D species (purple circle in Fig 1) and type 8 (AB448767) with a K2P value of 0.01 in ocular swab samples. Further analysis revealed that throughout the study period, the circulating strain of ocular Adenovirus in the Andaman Islands was D8 (Fig 1).

## Discussion

A hospital-based study was carried out among the population of the Andaman Islands. The suspected cases of acute keratoconjunctivitis were enrolled in the study, and clinical and molecular analyses were done to rule out the highly prevalent viral etiology among the study population. All the cases were found to be sporadic during the study period. So, the symptomatic observation revealed that eye redness 110 (87.30%) and watering 103 (81.75%) were the most prevalent symptoms, while chills and aches were not reported in our study. Apart from this, the molecular screening revealed the presence of adenoviruses responsible for 24% of all suspected cases. Besides this, the majority of cases had unilateral eye involvement during the acute phase, even though it was a highly contagious viral infection. In addition, the highly affected age group was 31-40, possibly due to the high social interaction and being more active in the public domain. If we talk about the gender distribution, males were found to have a greater association with infection compared with females.

According to earlier Indian studies, keratoconjunctivitis patients had a HAdV positive rate ranging from 13.8% to 66.6% [11-14]. Similar observations were obtained in our study, conducted in Andaman, with a 24% incidence rate. Tears (86%) and itching (71% of cases), rather than symptoms like fever, chills, and aches and pains in the eyes, were the most often reported symptoms in a prospective study from Madurai, India, which evaluated 106 patients suspected of having acute infectious conjunctivitis [15].

Using primers specific to the hexon region, this study confirmed the presence of HAdV type 8 from Group D in an ocular swab specimen. (Figure.1) Prior research conducted in India found that 78.6% of people in Pune and 100% in Pondicherry were affected by type 8 [16,17].

Furthermore, subclass A of the 7 HAdV subgroups (A to G) has been connected to the digestive system, while subgroups B and C are more common in the respiratory tract. However, conjunctivitis is most frequently brought on by HAdV subgroup D, while subgroup E is linked to both pulmonary and ocular conditions. Moreover, the subgroups known as HAdV-F and HAdV-G are used to classify the overwhelming majority of adenovirus-mediated gastroenteritis [18].

In clinical research, molecular epidemiology investigations are becoming more and more significant. Adenoviruses can cause a wide range of clinical illnesses, including conjunctivitis, gastroenteritis, hepatitis, myocarditis, and pneumonia. These disorders typically affect children under the age of five, and they usually go away on their own [18]. The current study discusses the association between adenoviruses and ocular mucosa, with a special emphasis on clinic-epidemiological aspects like eye redness and water discharge among the Andaman Island population, but none of the positive cases were associated with comorbidities.

The Andaman Islands are a popular destination for tourists worldwide, including those from mainland India. It is always expected that more people will move to these islands, leading to the transmission of several different viral illnesses. This study gives a foundational understanding of the prevalence and epidemiology of the human Adenovirus. However, this study has the limitation that the different tribal populations were not included in it and were restricted to the Andaman Islands, which are situated in the Bay of Bengal and far-flung from the mainland of India. Apart from this, due to the COVID-19 pandemic, we were unable to get more conjunctival swab samples of Keratoconjunctivitis suspected cases. Moreover, this study discusses the prevalence of adenoviral-mediated infection among the people who live on the Andaman Islands. Additional in-depth research must be conducted on such a highly contagious etiology, as well as its clinical-epidemiological pattern and the circulating strain among the susceptible population of this island and other surrounding remote islands.

## Conclusions

To sum up, this study reveals the prevalence of adenoviral infections among the inhabitants of the Andaman Islands. Besides this, the circulating strain was also detected, which emphasized that the Adenovirus is the cause of acute phase keratoconjunctivitis. However, there is a need for awareness regarding the ways of transmission of infection from one person to another. Besides this, regular screening programs of inhabitants of the Andaman Islands for adenoviral etiology from different infection sites are necessary to diagnose properly on time. Additionally, improvements in self-hygiene practices and proper sanitization can help reduce the rate of transmission of such contagious etiologies.
